# A novel machine learning-based approach for the computational functional assessment of pharmacogenomic variants

**DOI:** 10.1186/s40246-021-00352-1

**Published:** 2021-08-09

**Authors:** Maria-Theodora Pandi, Maria Koromina, Iordanis Tsafaridis, Sotirios Patsilinakos, Evangelos Christoforou, Peter J. van der Spek, George P. Patrinos

**Affiliations:** 1 Erasmus University Medical Center, Faculty of Medicine and Health Sciences, Department of Pathology, Bioinformatics Unit, Rotterdam, the Netherlands; 2 Laboratory of Pharmacogenomics and Individualized Therapy, Department of Pharmacy, School of Health Sciences, University of Patras, Patras, Greece; 3 The Golden Helix Foundation, London, UK; 4 Katharsis Technologies Inc., Chicago, USA; 5 Konstantopouleion General Hospital, Athens, Greece; 6 Zayed Center of Health Sciences, United Arab Emirates University, Al-Ain, United Arab Emirates; 7 Department of Pathology, College of Medicine and Health Sciences, United Arab Emirates University, Al-Ain, United Arab Emirates

**Keywords:** Machine learning, Computational approaches, Functional prediction, Pharmacogenomic variants

## Abstract

**Background:**

The field of pharmacogenomics focuses on the way a person’s genome affects his or her response to a certain dose of a specified medication. The main aim is to utilize this information to guide and personalize the treatment in a way that maximizes the clinical benefits and minimizes the risks for the patients, thus fulfilling the promises of personalized medicine. Technological advances in genome sequencing, combined with the development of improved computational methods for the efficient analysis of the huge amount of generated data, have allowed the fast and inexpensive sequencing of a patient’s genome, hence rendering its incorporation into clinical routine practice a realistic possibility.

**Methods:**

This study exploited thoroughly characterized in functional level SNVs within genes involved in drug metabolism and transport, to train a classifier that would categorize novel variants according to their expected effect on protein functionality. This categorization is based on the available in silico prediction and/or conservation scores, which are selected with the use of recursive feature elimination process. Toward this end, information regarding 190 pharmacovariants was leveraged, alongside with 4 machine learning algorithms, namely AdaBoost, XGBoost, multinomial logistic regression, and random forest, of which the performance was assessed through 5-fold cross validation.

**Results:**

All models achieved similar performance toward making informed conclusions, with RF model achieving the highest accuracy (85%, 95% CI: 0.79, 0.90), as well as improved overall performance (precision 85%, sensitivity 84%, specificity 94%) and being used for subsequent analyses. When applied on real world WGS data, the selected RF model identified 2 missense variants, expected to lead to decreased function proteins and 1 to increased. As expected, a greater number of variants were highlighted when the approach was used on NGS data derived from targeted resequencing of coding regions. Specifically, 71 variants (out of 156 with sufficient annotation information) were classified as to “Decreased function,” 41 variants as “No” function proteins, and 1 variant in “Increased function.”

**Conclusion:**

Overall, the proposed RF-based classification model holds promise to lead to an extremely useful variant prioritization and act as a scoring tool with interesting clinical applications in the fields of pharmacogenomics and personalized medicine.

**Supplementary Information:**

The online version contains supplementary material available at 10.1186/s40246-021-00352-1.

## Background

Various patient-specific factors (i.e., ethnicity, age, co-existing conditions, co-administered medications) have been associated with deviations between the expected and the observed effects owing to a specific medication. In addition, a significant percentage of these differential drug responses has been attributed to genetic variants located in genes involved in the processes of pharmacokinetics, pharmacodynamics, or even in genes coding for enzymes of the immune system (i.e., *HLA* genes), commonly described as pharmacogenes [1–3]. This genetically determined diversity of drug effects, as well as its exploitation toward tailoring the medication scheme is the primary focus of pharmacogenomics (PGx), and an integral component of personalized medicine. To this end, genotyping platforms, such as DMET™ plus by Affymetrix, can be used to detect well-characterized, common genetic variants [4]. Alternatively, next-generation sequencing (NGS), either whole exome sequencing (WES), whole genome sequencing (WGS), or even targeted resequencing, can be also used for this purpose, thus providing a more comprehensive idea of an individual’s genomic composition [5–7].

To date, 15% of the approved drugs by the EMA (European Medicines Agency) in the period 1995–2014 [8], and 7% of the drugs approved by the American Food and Drug Administration (FDA), are accompanied by pharmacogenomic recommendations [9]. Interestingly, relevant PGx biomarkers can be either germline variants in pharmacogenes, mostly single-nucleotide variations (SNVs) or copy number variants (CNVs), or somatic variants in cancer cells that affect tumor’s response to antineoplastic drugs, as well as epigenetic modifications of histones and DNA, which could potentially affect the drug response [3]. The effects of these PGx variants might range from altered drug exposure and hence modified efficacy or side effects, to idiosyncratic reactions [1–3].

The results of large-scale NGS analyses unravel several challenges, thus complicating the interpretation of the effects of PGx variants on protein function. For example, a large volume of novel, rare (minor allele frequency: MAF < 0.5%), population-specific SNVs, which could affect protein function has been detected within protein coding genes. These genes appear to be enriched in potentially damaging variants, owing to the combination of rapid population growth and weak action of purifying selection [10]. Similar observations were applied when focusing on 202 genes, the products of which are molecular targets for drug action [11]. Regarding the genes coding for phase I metabolic enzymes (CYPs) and drug transporters (UGT, ABC genes), the majority of the identified SNVs within these genes is ultra-rare (MAF < 0.1%) and non-synonymous, while variants that affect splicing sites or lead to loss of the termination codons, as well as nonsense changes are less common [12, 13]. Furthermore, the evaluation of organo anion transporter (OATP) transporter sequences provided by the Genome Aggregation Database (gnomAD) has underlined once again the importance of including novel, rare mutations (MAF < 1%) in the pharmacogenomic assays [14].

Taken together, NGS analyses have the potential to identify a very large number of PGx variants, most of which are novel, rare, and with no biochemical or clinical evidence for their impact on protein function. Performing functional expression assays for such large numbers of variants is not always feasible; hence, why the evaluation of predictions derived from in silico tools is an alternative approach to this end. The majority of computational methods used to assess the functional effect of variants in protein level are intended to distinguish neutral from deleterious variants, based on either a hypothesis (SIFT [15], PROVEAN [16]) or the evaluation of a set of properties, including secondary structure, functional sites, protein stability, and sequence conservation (PolyPhen-2 [17], MutPred [18], GERP++ [19]). More recently, a number of algorithms using unsupervised learning (Eigen, Eigen-PC [20]), as well as gene-level scores (LoFtool [21]) and ensemble approaches that integrate the predictions and training features of other tools have been also made available (DANN [22], Revel [23], MetaLR/MetaSVM [24]).

However, pharmacogenes and the respective PGx variants tend to differ from genes and variants implicated in disease. The suitability of features considered by the available algorithms is questionable, since genes coding for phase I and II metabolizing enzymes appear to be less conserved evolutionary [25], possibly due to their limited role in endogenous processes and the fact that only a mild modification of the pharmacokinetics and pharmacodynamics can lead to significant results [3]. Nevertheless, the development of an improved framework for the evaluation of pharmacogenomic variants, by combining different classifiers and appropriately adjusting their prediction thresholds, has led to promising results [26].

Herein, we propose a comprehensive model for the assessment of PGx variants by evaluating in silico protein prediction scores with the use of machine learning (ML), and thus highlighting the PGx variants that are most likely to alter the protein function and consequently have a PGx impact.

## Results

The current study focuses on exploiting publicly available and human variation data with well-defined protein-level functional consequences, to train a predictive model for the targeted classification of coding SNVs with regards to their protein function effects. The assigned protein function effect scores were based on the integration and assessment of in vitro biochemical assays, in vivo evidence and clinical data. Four different algorithms (AdaBoost, XgBoost, RF, multinomial logistic regression) were trained with a training set consisting of 190 variants, which  were located across 11 pharmacogenes and assessed with 5-fold cross validation. Finally, and as an attempt to utilize the method for real-world data, we assessed the applicability of the optimal model in NGS data, either whole genome or targeted sequencing data.

### Performance metrics for the machine learning models toward the functional assessment of PGx variants

The performance of the classifiers, which were constructed with variables recommended by the recursive feature elimination (RFE) method, was advantageous regardless of the limited sample size of the training set (*N* = 190 variants in 11 genes). More precisely, the metrics computed for the assessed machine learning models were as follows: random forest (RF) – accuracy: 0.85 (95% CI: 0.79, 0.90), area under the curve (AUC) = 0.92, area under the precision-recall curve (prAUC) = 0.73; AdaBoost – accuracy: 0.82 (95% CI: 0.76, 0.87), AUC: 0.91, prAUC: 0.72; XGBoost – accuracy: 0.80 (95% CI: 0.73, 0.85), AUC: 0.91, prAUC: 0.73; multinomial logistic regression – accuracy: 0.78 (95% CI: 0.72, 0.84), AUC: 0.93, prAUC: 0.74. Interestingly, multinomial logistic regression led to higher AUC and prAUC values compared to the tree-based approaches, while the achieved accuracy was the lowest among the assessed models.

**Fig. 1:**
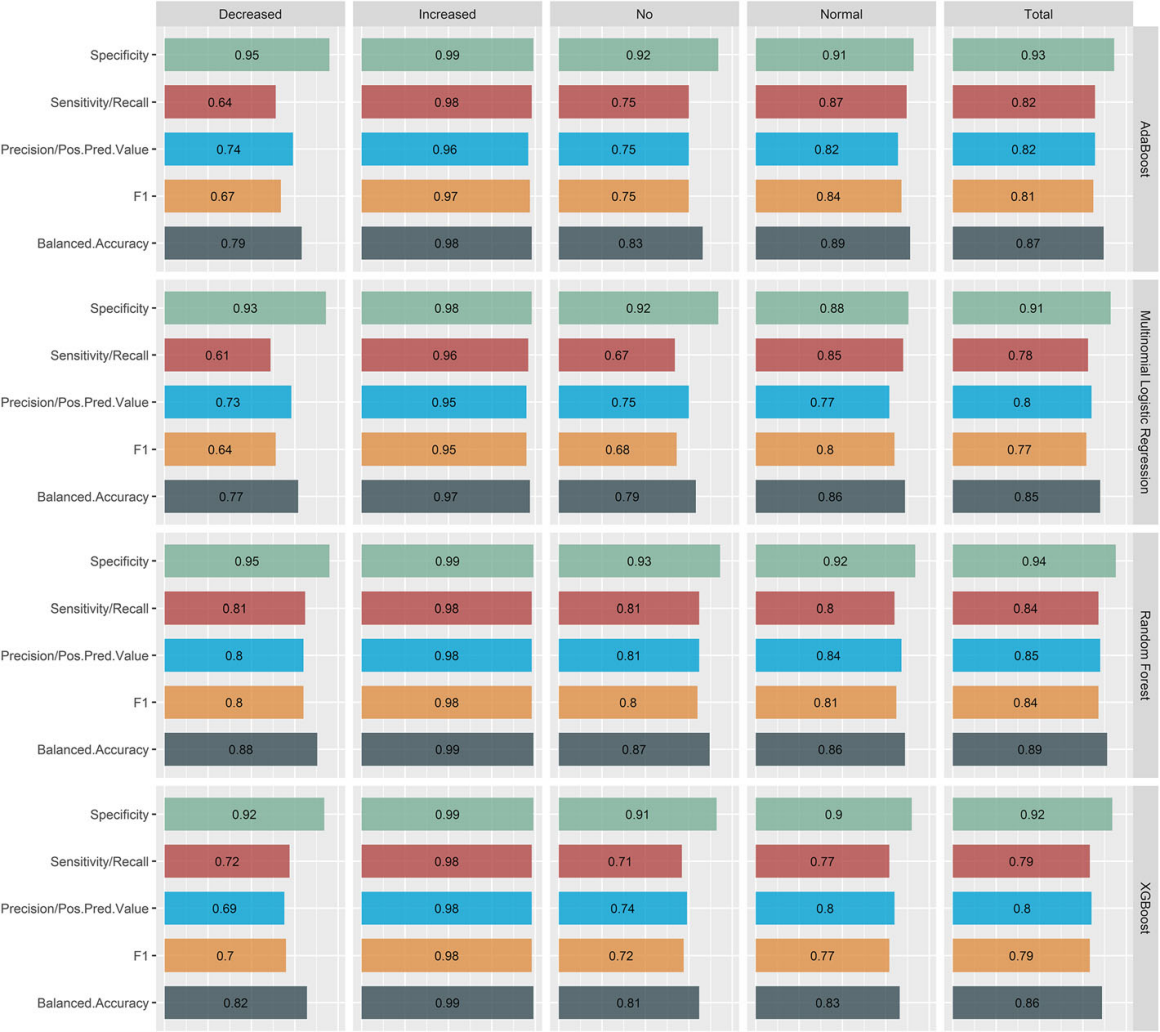
Metrics showing the performance of the different classifiers, namely AdaBoost, multinomial logistic regression, random forest, XGBoost. More specifically, the sensitivity, specificity, positive predictive value (pos.pred.value), precision, F1 metric (harmonic mean of precision and recall), and balanced accuracy of the classifiers are provided for each protein function effect class

RFs were selected as the final approach to be used for the described classification task, since the respective model presented overall improved performance (i.e., accuracy, sensitivity, specificity, and precision) across all four functional classes. Regarding the “Decreased function” variants, RFs were more sensitive and precise than the other assessed models, although AdaBoost achieved equal specificity values (Fig. 1). All models performed impressively well toward the “Increased function” category and led to very similar outcomes, while RFs appeared superior for the detection of “No function” variants and AdaBoost and multinomial logistic regression models were more sensitive for the “Normal function” class.

The selected machine learning model proved to be highly specific (≥ 92%) for all 4 functional variant classes, with lower, but still favorable values of sensitivity (8–98%), precision (80–98%) and balanced accuracy (86–99%). With regards to identifying variants that could lead to proteins with unchanged (normal), reduced, or no function, we observed the lowest values of the metrics.

**Fig. 2:**
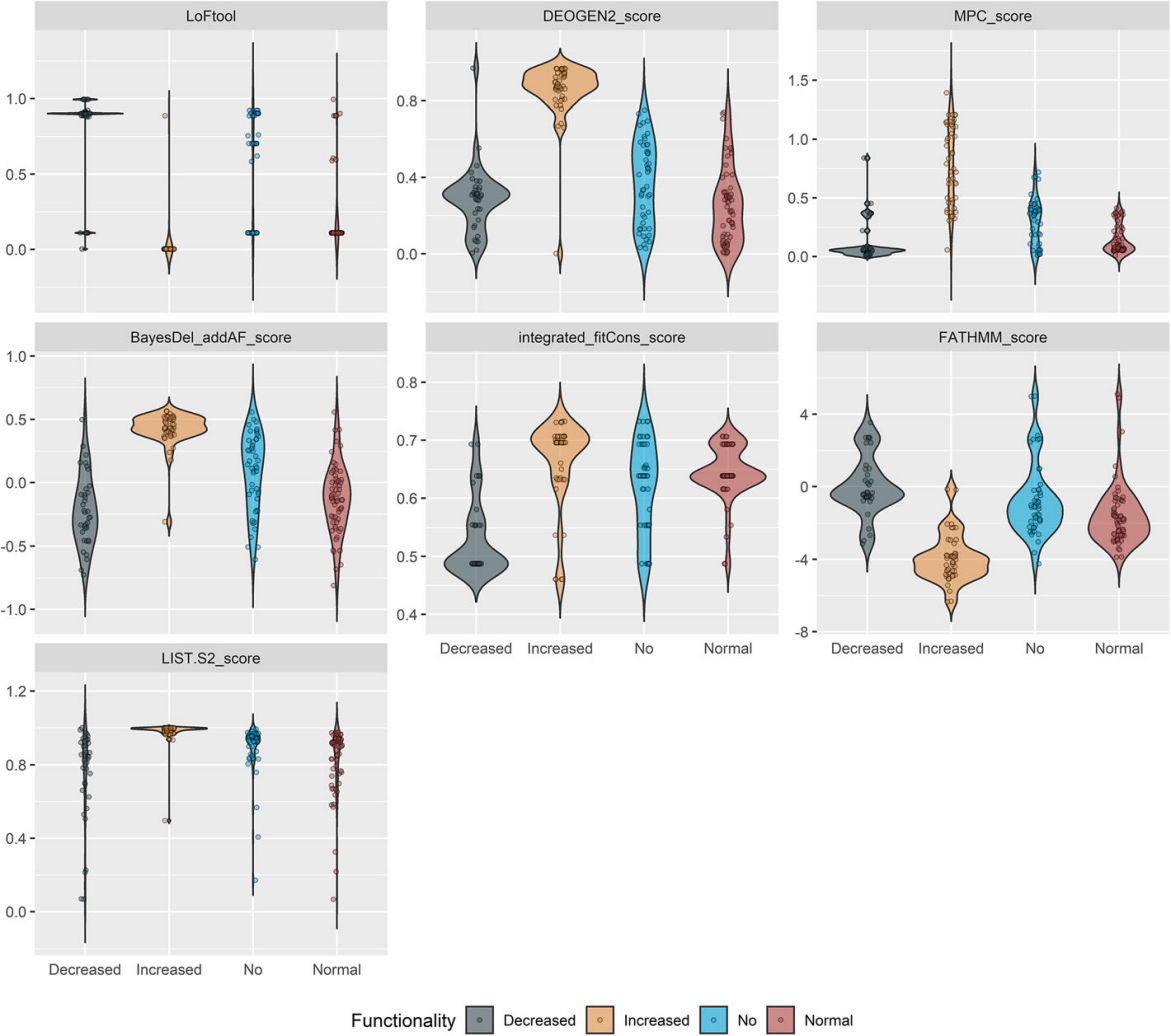
Violin plots depicting the distribution of each functionality class for certain in silico predictions tools, as suggested by the recursive feature elimination (RFE) procedure. The graphs show the distribution of values per each functionality class (“decreased,” “increased,” “no,” “normal”) for each one of the 7 RFE-suggested scores, which were used as the training features in the final classifier. These RFE-suggested scores are derived from LoFtool, DEOGEN2_score, MPC_score, BayesDel_addAF_score, integrated_fitCons_score, FATHMM_score, and LIST.S2_score

The model was characterized by a better performance for “Normal function” variants (sensitivity = 0.8, specificity = 0.92, precision = 0.84, balanced accuracy = 0.86), followed closely by “No” function variants (sensitivity = 0.81, specificity = 0.93, precision = 0.81, balanced accuracy = 0.87), and finally “Decreased function” variants (sensitivity = 0.81, specificity = 0.95, precision = 0.80, balanced accuracy = 0.88). Interestingly, the classifier performs extremely well for the category of “Increased Function” variants, in which case all computed metrics were above 98% (Fig. 1). To better explain the performance of the RF classifier with respect to four variant classes, the distribution of the training variants for the scores suggested by RFE and included in the classifier is provided in Fig. 2. The improved performance toward “Increased function” can be explained by the better definition of these variants compared to the rest classes (“No,” “Decreased,” and “Normal function”), which are characterized by a substantial extend of overlapping values, that could complicate their accurate classification.

We also attempted to assess the variables that could significantly affect the presented machine learning model. More specifically, when it comes to the variable importance, the highest-ranking positions were occupied by these features that RFE suggested as the most informative ones for the classification task. In the present instance, LoFtool emerged as the prominent for the categorization of a variant according to its effect on protein function (Figure S1, Supplementary Data).

### Comparing the RF model against other broadly used in silico tools

**Fig. 3:**
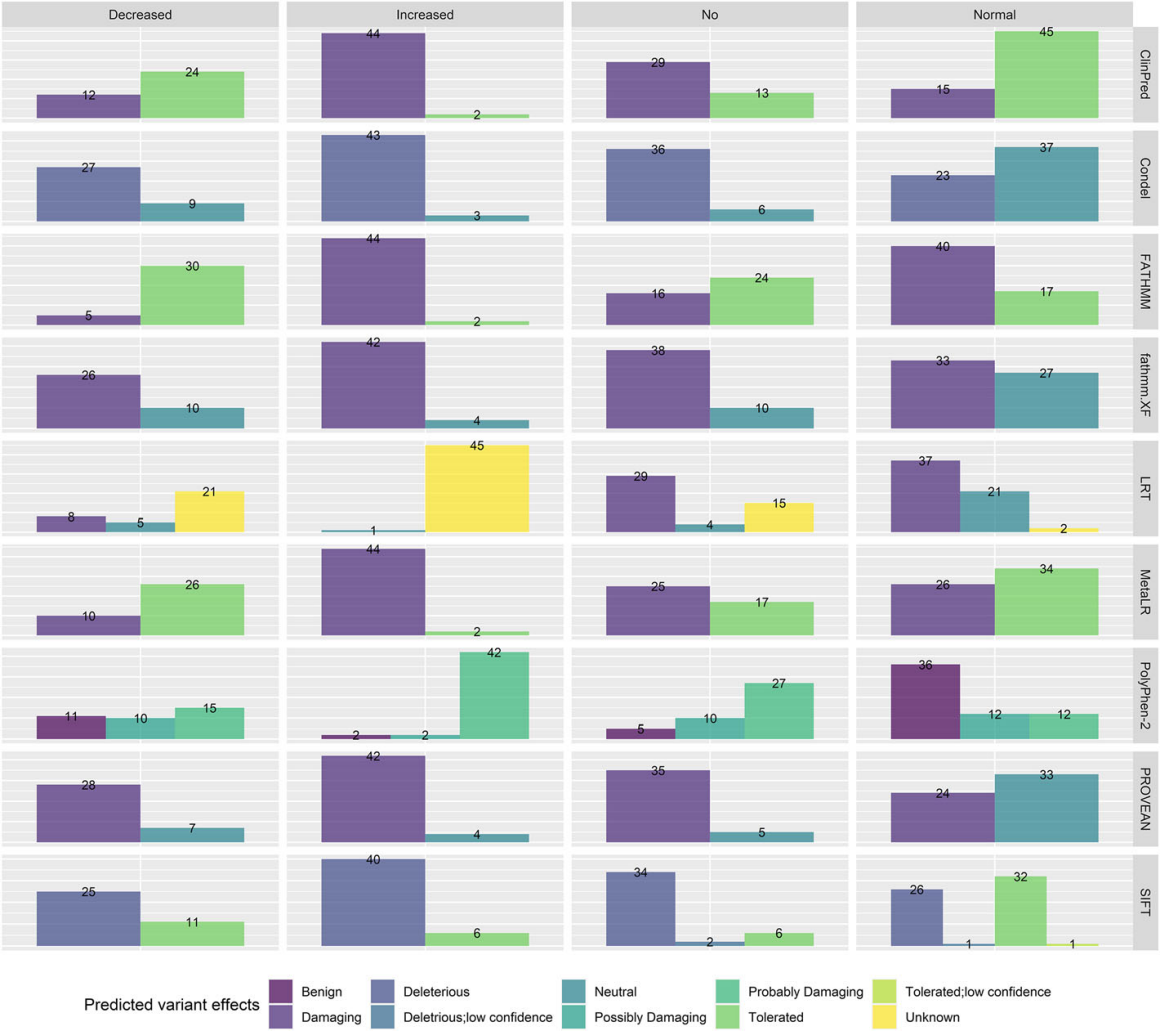
Classification of the variants included in our training set as based on broadly used in silico prediction tools. The columns show each functionality class (“decreased,” “increased,” “no,” and “normal”) and each row shows the distribution of variants within each class according to the predicted variant effect, as assigned per each in silico prediction tool. Predefined cut-off values for classification (as used in VEP and dbNSFP) are implemented. The scores are derived from ClinPred, Condel, FATHMM, fathmm.XF, LRT, MetaLR, Polyphen-2, PROVEAN, and SIFT

As a further step, we assessed how different, commonly used functionality prediction algorithms would classify the 190 variants that were included in the final training set. Toward this end, ClinPred [27], Condel [28], FATHMM [29], Fathmm-XF [30], LRT [31], MetaLR [24], PolyPhen-2 [32], PROVEAN [16], and SIFT [33] were selected and the corresponding predictions, as provided by VEP, are presented in Fig. 3. Of these scores, only FATHMM-XF can be also applied to non-coding variants, while the rest are intended for use in coding, non-synonymous SNVs. In addition, ClinPred, Fathmm, and MetaLR classify variants as either “Tolerated” or “Damaging”; Condel as “Neutral” or “Deleterious”; FATHMM-XF and PROVEAN as “Neutral” or “Damaging”; LRT as “Deleterious,” “Neutral,” or “Unknown’; PolyPhen-2 as “Benign,” “Possibly Damaging,” or “Probably Damaging”; and, finally, SIFT as “Tolerated,” “Tolerated with low confidence,” “Deleterious with low confidence,” and “Deleterious.” As a first observation, none of these tools covers variants that could lead to gain-of-function. Although this functionality is provided by B-SIFT [34], it is not available through VEP, and thus, it could not be included in the analysis. Regarding increased function variants, all algorithms, except LRT categorize these variants as either “Damaging” or “Deleterious.” In addition, there is apparent discordance among the tools’ classification of “decreased” and “normal” function variants, while most algorithms can identify variants leading to non-functional proteins.

### Application of the machine learning model in NGS data

#### First case study (WGS data)

To further demonstrate the prediction performance of the final RF model, we tested its applicability in “unseen” NGS data, namely those data that have not been previously used to train the machine learning algorithm. We first tested its applicability in WGS data from a patient diagnosed with coeliac disease. From this process, 1808 variants, including 3 novel, within the 10 pharmacogenes of interest (*DPYD*, *CYP2C19*, *CYP2C9*, *SLCO1B1*, *NUDT15*, *RYR1*, *CYP2B6*, *UGT1A1*, *CYP2D6*, *TPMT*) were identified. Of these, only six missense variants had adequate information, i.e., no missing values in the incorporated functional prediction scores, to be to be further processed by the RF model. With regards to the observed allele frequency, four were found to be common (rs1801159, rs2306283, rs4149056, rs35364374), one had intermediate frequency (rs3745274), and one was ultra-rare (rs762454967) with MAFs based on GnomAD genomes. Of these 1808 analyzed variants, we did not identify any variants categorized as loss-of-function variants (LoF).

Table 1 presents these variants, alongside with their predicted functional impact, as defined by the majority vote of the individual decision trees. For example, a random forest containing 1000 distinct decision trees was built. If most of those votes recommend that the variant belongs to “No function” variants, then this is the class that is attributed to the variant. In addition, the probability of being classified in each class, as based on the votes of all trees of the random forest built, is also provided (Table 1).

**Table 1:** Classification outcomes (prediction and probabilities) for WGS data using the final RF model. The predicted class is determined based on a majority vote from the individual decision trees of the random forest classifier, while the presented probabilities depict the corresponding percentage of decision trees voting toward a functional class

Location (GRCh38)	Allele	Existing variation	SYMBOL	HGVSc	GnomAD AF (%)	Predicted class	Probability of attributed class
1:97515839-97515839	C	rs1801159, CM033371, COSV64593269	*DPYD*	ENST00000370192.8:c.1627A>G	18.49%	Normal	0.96
12:21176804-21176804	G	rs2306283, CM043776, COSV57012766	*SLCO1B1*	ENST00000256958.3:c.388A>G	53.33%	Normal	0.66
12:21178615-21178615	C	rs4149056, CM043777, COSV57010105	*SLCO1B1*	ENST00000256958.3:c.521T>C	11.95%	Decreased	0.88
19:38492540-38492540	T	rs35364374	*RYR1*	ENST00000359596.8:c.6178G>T	4.95%	Increased	0.38
19:38499641-38499641	A	rs762454967, CM140865	*RYR1*	ENST00000359596.8:c.7034G>A	0.00%	Increased	0.80
19:41006936-41006936	T	rs3745274, CM130453, CS080663, COSV57843253	*CYP2B6*	ENST00000324071.10:c.516G>T	28.44%	Decreased	0.72

This computational process led to the confirmation of 2 missense variants (located within the *SLCO1B1* and *CYP2B6* genes, respectively) that could potentially lead to proteins with decreased functionality and 1 missense variant classified as “increased function” (located in *RYR1*). The remaining two variants were predicted to lead to no changes in the protein function (i.e., normal). The rest of the PGx variants had a high rate (over 85%) of missing values in the features of interest and were mostly (*N* = 1765 out of 1803; 97.89%) located within intronic regions (Figure S2, Supplementary Data). The latter were followed by variants in 3′ prime UTRs (*N* = 20; 1.11%), missense (*N* = 6; 0.33%), and synonymous (*N* = 11; 0.61%) variants. Interestingly, *DPYD* which encodes for a drug-metabolizing enzyme accumulated more than 1000 intronic variants.

Regarding the potential clinical actionability of these 6 variants (rs1801159, rs2306283, rs4149056, rs35364374, rs3745274, and rs762454967), we retrieved additional information from the PharmGKB database. rs1801159 and rs2306823 were not associated with any predicted changes in the protein function or changes in the dosing guidelines (i.e., normal, or low-level changes respectively). However, changes in treatment were recommended for individuals with the rs4149056 variant genotype, while also stating that any additional risk factors should be considered for statin-induced myopathy. Moreover, rs3745274 carried multiple levels of CPIC evidence, for a variety of drugs such as efavirenz, nevirapine, propofol, imatinib, cyclophosphamide, doxorubicin, mitotane, methadone, and 3,4-methylenedioxymethamphetamine. No PGx clinical information could be retrieved for rs35364374 and rs762454967 within *RYR1*, which were both predicted as “increased function” variants.

### Second case study (targeted PGx sequencing data)

The second case study consisted of targeted PGx sequencing data from 304 individuals of Greek origin and diagnosed with psychiatric disorders. Interestingly, 343 variants were identified, covering 10 pharmacogenes (*DPYD*, *CYP2C19*, *CYP2C9*, *CYP2C8*, *SLCO1B1*, *NUDT15*, *CYP2B6*, *UGT1A1*, *CYP2D6*, *TPMT*), 18 of which were attributed a SO consequence indicative of LoF variants. More specifically, we found 11 “frameshift,” 6 “stop gained,” and 1 “start lost” variants. None of these variants was assessed by the RF model, owing to the high levels of missing values (mean, 77% missing values in the scores of interest). The remaining variants were mostly missense (*N* = 205) or synonymous (*N* = 107) (Figure S3). According to GnomAD genome frequencies in the general population (AF), which were available for 88 of these variants, the dataset was enriched for “ultra-rare” variants (MAF ≤ 0.1%) (*N* = 42), followed by “rare” (0.1% ≤ MAF < 1%) (*N* = 18), “low frequency” (1% ≤ MAF < 5%) (*N* = 14), “common” (MAF ≥ 10%) (*N* = 8), and “intermediate” (5% ≤ MAF < 10%) (*N* = 6) variants.

**Fig. 4:**
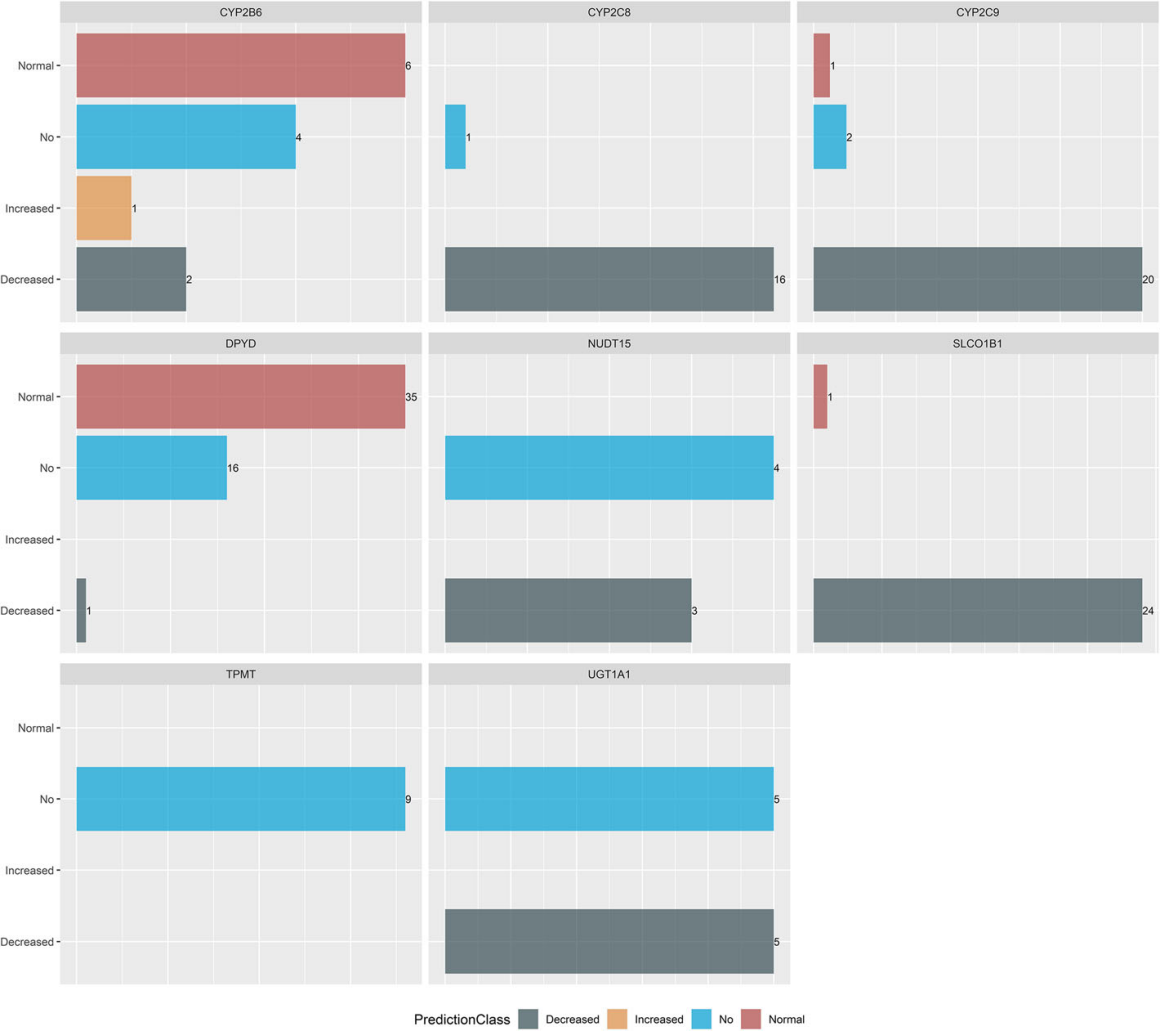
Protein function predictions based on the final RF model after assessment of the targeted PGx sequencing data from a Greek cohort of 304 individuals. The functionality class for the PGx variants, as processed by the RF model, is depicted per each pharmacogene of interest. The numbers denote the totaling number of variants within each pharmacogene per each function class

The dataset of 343 variants included 195 known and 148 novel variants, of which 86 novel and 70 known PGx variants (156 in total) were evaluated by the final RF model (data available upon request). The evaluated variants were mostly missense (i.e., 149 “missense,” 7 “missense/splice region”). Of these, 71 variants led to “Decreased” function proteins, 41 variants to “No” function proteins, 1 variant in “Increased” function protein, and 43 variants have no effect on protein functionality (i.e., “normal” function) (Fig. 4).

To further estimate the potential clinical actionability of the 156 PGx variants, as evaluated by the RF model, additional clinical and variant information was retrieved from PharmGKB. rs1801159, rs1801158, rs2297595, and rs1801160 were not associated with any predicted changes in the protein function, according to the variant annotation by PharmGKB, which constitutes an observation in concordance with the assigned prediction classes by the RF model (i.e., “normal” function class). Moreover, rs67376798 was associated with decreased catalytic activity based on evidence from PharmGKB, thus further confirming the prediction class of the RF model (i.e., “decreased” function class). Similar observations were applied for the variants, namely rs4149056, rs116855232, and rs3745274, for which the following prediction classes were assigned by the RF model: “decreased,” “no,” “decreased,” respectively. PharmGKB provides multiple levels of clinical evidence for these variants, the majority of which were associated with decreased protein activity, therefore confirming the presented model results.

## Discussion

Conventional genetic testing and clinical guidelines focus solely on a small number of well-studied variants or star alleles in pharmacogenes, while the application of NGS techniques provides the possibility to detect a much wider range of (PGx) variants. Recent studies have demonstrated that coding variants are rare, population-specific and a significant proportion of them could potentially affect the protein product (based on in silico assays and metrics) [10–14]. At the same time, the role of copy number variants (CNVs) within pharmacogenes [35], as well as variants in non-coding regions, is gaining more attention, with more than 90% of the polymorphisms detected in GWAS pharmacogenomic studies being non-coding [36]. Owing to the limited number of thoroughly documented PGx variants and the incredibly large number of identified genetic mutations that should be experimentally validated, the initial evaluation of these variants found must be performed via the use of in silico tools.

The study’s main aim was the assessment of the utility of in silico-derived scores, commonly used for variant annotation, toward the characterization of the potential protein function effects of SNVs identified within pharmacogenes. Among the assessed algorithms (AdaBoost, XGBoost, RF, multinomial logistic regression), RF presented superior performance and was selected as the final classifier. RFs have been also proven to be robust in the presence of outliers or noise, effective, even without configuration, and useful in cases where the number of available “-omics” data is limited, when compared to the number of available variables [37, 38].

The final classifier required minimum hyperparameter tuning and integrated 7 scores, stand-alone or ensemble ones, and 2 custom created variables. The overall accuracy was equal to 0.85 (95% CI: 0.79, 0.90), with an area under the curve of 0.92 and an area under the precision-recall curve (PR AUC) of 0.73. The by-class performance for variants of Normal, Decreased, and No Function classes is efficient enough, although there is still room for improvement, especially in terms of sensitivity (0.80, 0.81, and 0.81 respectively). Interestingly, the model appears to be efficient, given the fact that most of the incorporated features are used to distinguish between damaging and benign variants, specifically when it comes to identifying increased function SNVs. Furthermore, LoFtool, an approach that evaluates the tolerance of a gene to loss-of-function mutations, emerged as the most significant determinant of the classification task. The superior performance of the model in identifying “Increased” Function PGx variants, combined with the observation that this specific class in the training dataset represents only two pharmacogenes, might partially justify the importance of the variable.

Although there is limited published work in this specific area, the possibility of using PGx variants so as to develop classification tools has been previously explored, without however progressing any further due to the limitations and difficulties that accompany this field [39]. Firstly, the most frequently examined properties in such classifications tools are the degree of evolutionary conservation, which is observed in lower levels in pharmacogenes [3] and therefore its usefulness is debated by a series of studies [26, 39, 40], as well as parameters regarding the structure of the respective proteins, which have been observed to lead to small increases in the efficiency of the classifiers produced [39]. Overall, such factors could influence the quality of the output results in classification models, as the one presented herein.

In addition, the training sets used to train computational models are usually comprised of common polymorphisms against variants (mostly SNVs) related to disease-causality, while in terms of drug response, the modifying effect of common genomic variants cannot be ruled out. Moreover, the resulting scores evaluate the pathogenic potential of the examined variants and classify them into two usually categories according to certain applied thresholds. In contrast, PGx researchers usually focus on the induced change in protein function, which can be distinguished at several levels (e.g., increase, decrease, no change, complete loss of activity), while the differential drug response is not a disease, but a phenotype that occurs under specific conditions (i.e., administration of a specific drug).

For example, in a recent study, the adaptation of the proposed classification thresholds and the subsequent integration of selected algorithms, which could provide optimal results for the creation of a comprehensive score, led to a tool with exceptional sensitivity and specificity [26]. However, this work focused exclusively on the distinction between loss-of-function and neutral variants, hence ignoring PGx variants that would result in a protein product of increased activity, and which are of interest in PGx field.

The novelty of our recommended approach lies in the computational “design” of the classifier Specifically, starting from a VEP annotated .vcf file as the input, the classifier quickly leads to a list of PGx variants that could harbor a protein function effect and hence a potential clinical PGx impact. Unlike disease-related variants, there is no state-of-the-art procedure so far—to the best of our knowledge—which can be used to interpret variants implicated in drug response [41]. Taken together, the originality of the presented model lies both in the variant analysis process automatization and the incorporation of available in silico scores for the evidence-based assessment of pharmacovariants.

Given the challenges and implications for the prediction of functional impact of PGx variants, as well as the complexity of the involved biological processes [42], the findings of this study should be interpreted with caution. For example, discrepancies have been observed not only among different algorithms, or between in silico predictions and in vitro activity [43], but also when comparing in vitro and in vivo observations. A characteristic example is that of CYP2D6*35, which has not been associated with reduced activity, despite the experimental evidence of reduced hydroxylation capacity of tamoxifen [44, 45]. Moreover, researchers should keep in mind that the same variant may affect the response to different drugs in different ways. For example, although the CYP2C8*10 and CYP2C8*13 alleles have been found to affect the *N*-deethylation of amodiacin, the hydroxylation of paclitaxel—which is also metabolized by CYP2C8—remains unaffected [46].

As mentioned earlier, the presented model has demonstrated promising results, despite the limitations of this computational research field. However, there is still space for further improvements toward a more efficient and robust version of the presented model. More specifically, it would be useful to examine and compare the performance of other machine learning (ML) approaches, supervised or not. Furthermore, significant advantages are expected to emerge from the collection and curation of larger training sets, consisting of larger numbers of variants and covering an additional number of pharmacogenes. Furthermore, the computed metrics demonstrate a difficulty in distinguishing between normal and decreased/no function variants, thus making debatable the suitability of the used features for the characterization of these PGx variants.

Moreover, the integration of CNVs and non-coding variants, although promising, is often difficult to achieve owing to the limited number of available tools and approaches for CNV calling and for the functional assessment of non-coding variants. Emphasis should be also placed on the creation of well-characterized sets of PGx variants at the level of protein effects, both laboratory and clinical, as well as on the improvement of the existing databases to facilitate the export of the requested information. In addition, researchers should consider that an individual does not carry just one variant in one pharmacogene; therefore, the combination of PGx variants is often what results in the overall difference in drug response [25]. To this end, since the contribution of various factors to the response to a given drug is non-debatable, a more comprehensive approach through systemic genomics would be particularly useful, thus incorporating a variety of different -omics data [47].

## Conclusions

The novelty of the computational model presented herein lies in the fact that a ML approach was used to classify PGx variants, particularly novel and rare variants, by consequently assigning a protein activity prediction. Overall, the presented model prioritizes annotated PGx variants in different variant effect classes and then assigns a protein function classification after stringent computational assessment and ML processes. Its utility was further showcased by using two real-life datasets to further support the applicability of this model as a clinical support decision tool. Indeed, a validated, methodical prioritization of the multitude of genomic variants stemming from NGS analyses, as the one presented herein, has the potential to positively contribute toward the large-scale clinical application of pharmacogenomics and facilitate the translation of a patient’s genomic profile into actionable clinical information.

## Methods

### Collecting the training data

An appropriate training set of variants was manually curated using the PGx gene-specific information tables, created under the collaboration between PharmGKB and CPIC and was subsequently supplemented by additional variants from PharmVar [48]. This training set consists of 262 variants located across 12 pharmacogenes, with well-defined protein-level functional consequences, as based on the integration and assessment of in vitro biochemical assays, in vivo evidence and clinical observations. After careful data examination and owing to the high percentages of missing values, 190 variants within 11 pharmacogenes (Table S1, Supplementary Data) remained and were used as our training set. The observed functionality is classified into 5 levels (excluding Unknown/Uncertain function): “Increased”, “Normal”, “Possibly Decreased”, “Decreased” and “No function”. However, owing to the limited number of observations harboring the levels of “Possibly Decreased” and “Decreased” functions and after careful examination of the available information for those categories, these two levels were combined in one class (Decreased function) (Table 2).

**Table 2:** Description of the protein function effect classes of PGx variants, which are used as the training data for the final RF model. The functionality class is split in the following classes (“decreased,” “increased,” “no,” “normal”), the number of the respective PGx variants per class is also provided, as well as which pharmacogenes are incorporated per each class

Functionality class	Number of variants	Representation of genes
Decreased	36	*DPYD*, *CYP2C19*, *CYP2C9*, *SLCO1B1*, *RYR1*, *CYP2B6*, *UGT1A1*, *CYP2D6*
Increased	46	*RYR1*, *CYP2B6*
No	48	*CYP2C19*, *CYP2C8*, *DPYD*, *CYP2C9*, *NUDT15*, *CYP2B6*, *CYP2D6*, *TPMT*
Normal	60	*DPYD*, *CYP2C9*, *SLCO1B1*, *CYP2B6*, *CYP2D6*

### Variant annotation

The curated set of pharmacogenomic variants was annotated using the web interface of Ensembl’s variant effect predictor (VEP) tool, for the GRCh38 human assembly, as well as the 4.1.a version of the dbSNFP database [49], which is also provided through VEP. The majority of the retrieved information is available for variants located within protein coding regions and includes: a detailed characterization at a protein level (i.e., database identifiers, codons, amino acids, coordinates, protein domains, computational scores, etc.), overlapping known variants, observed frequencies in different populations (i.e., via the 1000 Genomes Project, the genome Aggregation Database, the Exome Aggregation Consortium data and the Exome Sequencing Project), any related phenotypes (e.g., OMIM, Orphanet, GWAS catalog) or clinical significance (ClinVar), as well as literature references [50]. Furthermore, the attributed consequence, described by using terms as developed in collaboration with Sequence Ontology (SO) [51], and the corresponding impact of a variation are also provided.

Regarding the retrieved frequency data, variants were classified as “common” if the minor allele frequency (MAF) was equal or above 10% (MAF ≥ 10%) and as “intermediate” if the MAF ranged between 5 and 10% (5% ≤ MAF < 10%). Variants were classified as “low frequency” if the MAF ranged between 1 and 5% (1% ≤ MAF < 5%), while “rare” variants included these variants of which the MAF was between 0.1 and 1% (0.1% ≤ MAF < 1%). Finally, variants were classified as ultra-rare if the MAF was equal or below 0.1% (MAF ≤ 0.1%).

Features and variants with a high percentage of missing values (≥ 40%) were excluded, while the remaining values were imputed by using k-nearest neighbors algorithm (kNN) [52] with default values for k-neighbors (equal to 5) and inverse weighted mean Gower distances [53]. In addition, a step of backwards variable selection through RFE using Bagged Trees was performed, which recommended the use of 7 out of the 45 variables (LoFtool [21], DEOGEN2_score [54], MPC_score [55], BayesDel_addAF_score [56], integrated_fitCons_score [57], FATHMM_score [29], LIST.S2_score [58]). Furthermore, two binary variables were constructed and included in the analysis: one indicating whether the variant was located within a protein functional domain (according to InterPro [59] annotation) and one representing high impact SO consequences (splice acceptor or donor variants, stop gained, frameshift variants, stop or start lost), enriched for loss-of-function (LoF) changes, as defined by MacArthur and coworkers (2012) [60].

### Training of the machine learning model

All preprocessing and ML-related analyses described in this work were performed using the R language for statistical programming (version 4.0.2) [61]. To exploit the abilities of the abovementioned features toward explaining potential protein function effects of variants derived from NGS analyses, a variety of tree-based methodologies was assessed, alongside with a special case of a neural network acting in a multinomial logistic regression manner. More specifically, random forests [62, 63], multi-class AdaBoost [64, 65], XGBoost [66], and a neural network striped from its hidden layers and activation functions (multinomial logistic regression) [67, 68] were used via the caret package [69]. For the selected tree-based models, hyperparameters were tuned based on the optimization of the accuracy metric, while in multinomial logistic regression, the default parameters were used (Table S2, Supplementary Data).

### Evaluation of the machine learning models

The predictive performance of the created models was assessed via the 5-fold cross validation (CV) method. During n-fold CV, the data are divided to create *n* equal-sized subsets; *n*-1 of these are used to train a model and the remaining 1 is used to test its performance. This process is repeated n times, until all subsets have been used to test the model, while the computed metrics in each iteration are averaged. More specifically, the metrics of interest include the accuracy, precision, sensitivity (true Positive rate), specificity (True Negative rate), balanced accuracy (average of precision and recall), and the F-measure (harmonic mean of precision and recall). Since this was a multi-class task, all metrics were computed for each class separately (according to the one-vs-all method), and the performance of the model was calculated using the corresponding weighted average values for each metric. Furthermore, a random forest classifier was trained with the total of 47 features and used to evaluate their predictive importance.

### Testing the applicability of the final machine learning model

To further demonstrate the applicability of the machine learning model, we applied the classifier in data derived from NGS analyses. Τo this end, variant call format (.vcf) files comprised of the results from (i) a WGS analysis of a single individual of Greek origin diagnosed with coeliac disease, and (ii) a targeted pharmacogene sequencing analysis of 304 individuals of Greek origin diagnosed with psychiatric diseases [70]. Firstly, the provided variants were annotated, using the web interface of ensemble VEP tool, while the resulting data were preprocessed to select only these identified in the transcripts of interest. Then, these annotation data were used as an input to our final RF model and the corresponding prediction functionality classes and prediction probabilities were provided.

Last, clinical and variant annotations found in PharmGKB (https://www.pharmgkb.org) were also curated to extract clinically relevant information for the PGx variants either assessed or missed by the presented RF model.

## Supplementary Information

**Additional file 1: Figure S1.** Annotation features examined as training variables in the machine learning model for the functional assessment of pharmacogenomics variants. These features are ranked according to their suggested interpretational significance from least (bottom) to most important (top). **Figure S2.** Distribution of PGx variants identified in the WGS data (first case study) that were not processed owing to many missing values. The graph presents the number of PGx variants, by gene, that were not processed any further by the machine learning model, according to the VEP consequence (i.e., 3’ UTR variant, intronic variant, missense variant, splice region variant and synonymous variant). The pharmacogenes are color-coded according to the corresponding PGx group: genes encoding drug metabolizing enzymes or genes encoding drug transporters or other non-metabolizing enzymes. Figure **S3**. Sequence ontology consequences for the identified PGx variants, as derived from a Greek cohort of 304 individuals with psychiatric disorders (second case study). 343 PGx variants within the pharmacogenes of interest were identified in this cohort. Amongst the consequences are ‘frameshift’, ‘missense’, ‘missense or splice region’, ‘splice region’, ‘start lost’, ‘stop gained’ and ‘synonymous’ variants. **Supplementary Table S1.** List of the represented pharmacogenes, which were included in the training dataset of the assessed machine learning models (AdaBoost, Multinomial logistic regression, Random Forest, XGBoost). **Table S2.** Summary of the parameters and metric values for the tree-based models (AdaBoost, Random Forest, XGBoost), as tested in the present study. Parameters denoted with an asterisk (*) were tuned according to the achieved accuracy.

## Abbreviations

SNVs: Single-nucleotide variations
PGx: Pharmacogenomics
NGS: Next-generation sequencing
WES: Whole exome sequencing
WGS: Whole genome sequencing
EMA: European Medicines Agency
FDA: Food and Drug Administration
CNVs: Copy number variants
MAF: Minor allele frequency
RF: Random forest
AUC: Area under the curve
prAUC: Area under the precision-recall curve
