# Supplementary material for: A novel machine learning-based approach for the computational functional assessment of pharmacogenomic variants
Source: Hum Genomics. 2021 Aug 9;15:51. doi: 10.1186/s40246-021-00352-1 (PMC8351412; doi:10.1186/s40246-021-00352-1)
Supplement: Supplementary file 1 — Additional file 1: Figure S1. Annotation features examined as training variables in the machine learning model for the functional assessment of pharmacogenomics variants. These features are ranked according to their suggested interpretational significance from least (bottom) to most important (top). Figure S2. Distribution of PGx variants identified in the WGS data (first case study) that were not processed owing to many missing values. The graph presents the number of PGx variants, by gene, that were not processed any further by the machine learning model, according to the VEP consequence (i.e., 3’ UTR variant, intronic variant, missense variant, splice region variant and synonymous variant). The pharmacogenes are color-coded according to the corresponding PGx group: genes encoding drug metabolizing enzymes or genes encoding drug transporters or other non-metabolizing enzymes. Figure S3. Sequence ontology consequences for the identified PGx variants, as derived from a Greek cohort of 304 individuals with psychiatric disorders (second case study). 343 PGx variants within the pharmacogenes of interest were identified in this cohort. Amongst the consequences are ‘frameshift’, ‘missense’, ‘missense or splice region’, ‘splice region’, ‘start lost’, ‘stop gained’ and ‘synonymous’ variants. Supplementary Table S1. List of the represented pharmacogenes, which were included in the training dataset of the assessed machine learning models (AdaBoost, Multinomial logistic regression, Random Forest, XGBoost). Table S2. Summary of the parameters and metric values for the tree-based models (AdaBoost, Random Forest, XGBoost), as tested in the present study. Parameters denoted with an asterisk (*) were tuned according to the achieved accuracy. [file 40246_2021_352_MOESM1_ESM.docx]

**Supplemental data**

**A novel machine learning-based approach for the computational functional assessment of pharmacogenomic variants**

**Maria-Theodora Pandi ^1,#^, Maria Koromina ^2,3,#^, Iordanis Tsafaridis ^4^, Sotirios Patsilinakos ^5^, Evangelos Christoforou ^1^, Peter J van der Spek ^1^, George P. Patrinos ^2,6,7^**

^1^ Erasmus University Medical Center, Faculty of Medicine and Health Sciences, Department of Pathology, Bioinformatics Unit, Rotterdam, the Netherlands; ^2^ Laboratory of Pharmacogenomics and Individualized Therapy, Department of Pharmacy, School of Health Sciences, University of Patras, Patras, Greece; ^3^ The Golden Helix Foundation, London, UK; ^4^ Katharsis Technologies Inc.; ^5^ Konstantopouleion General Hospital, Athens, Greece; ^6^ Zayed Center of Health Sciences, United Arab Emirates University, Al-Ain, United Arab Emirates; ^7^ Department of Pathology, College of Medicine and Health Sciences, United Arab Emirates University, Al-Ain, United Arab Emirates; ^#^: These authors contributed equally to this work


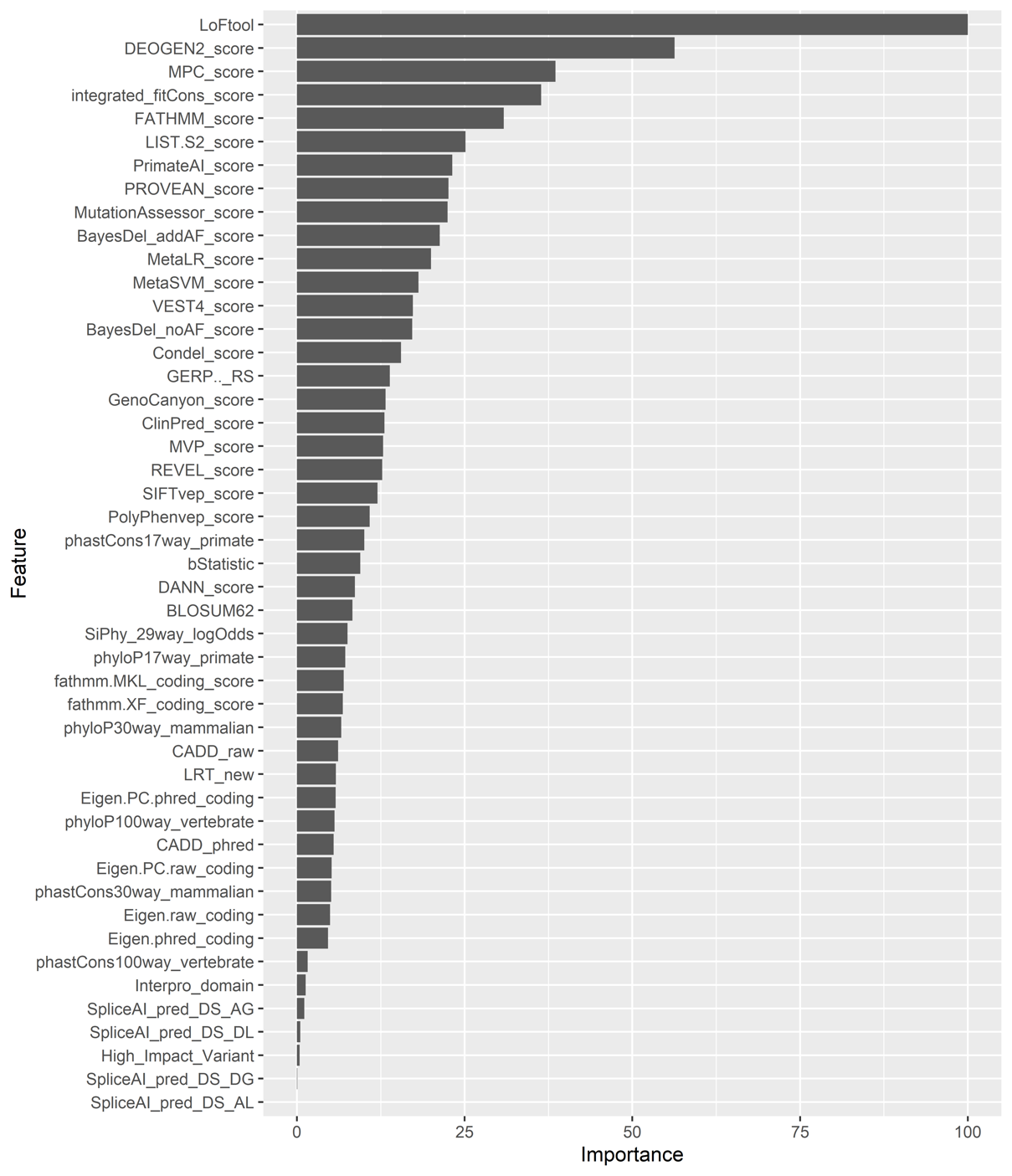


**Figure S1.** Annotation features examined as training variables in the machine learning model for the functional assessment of pharmacogenomics variants. These features are ranked according to their suggested interpretational significance from least (bottom) to most important (top).


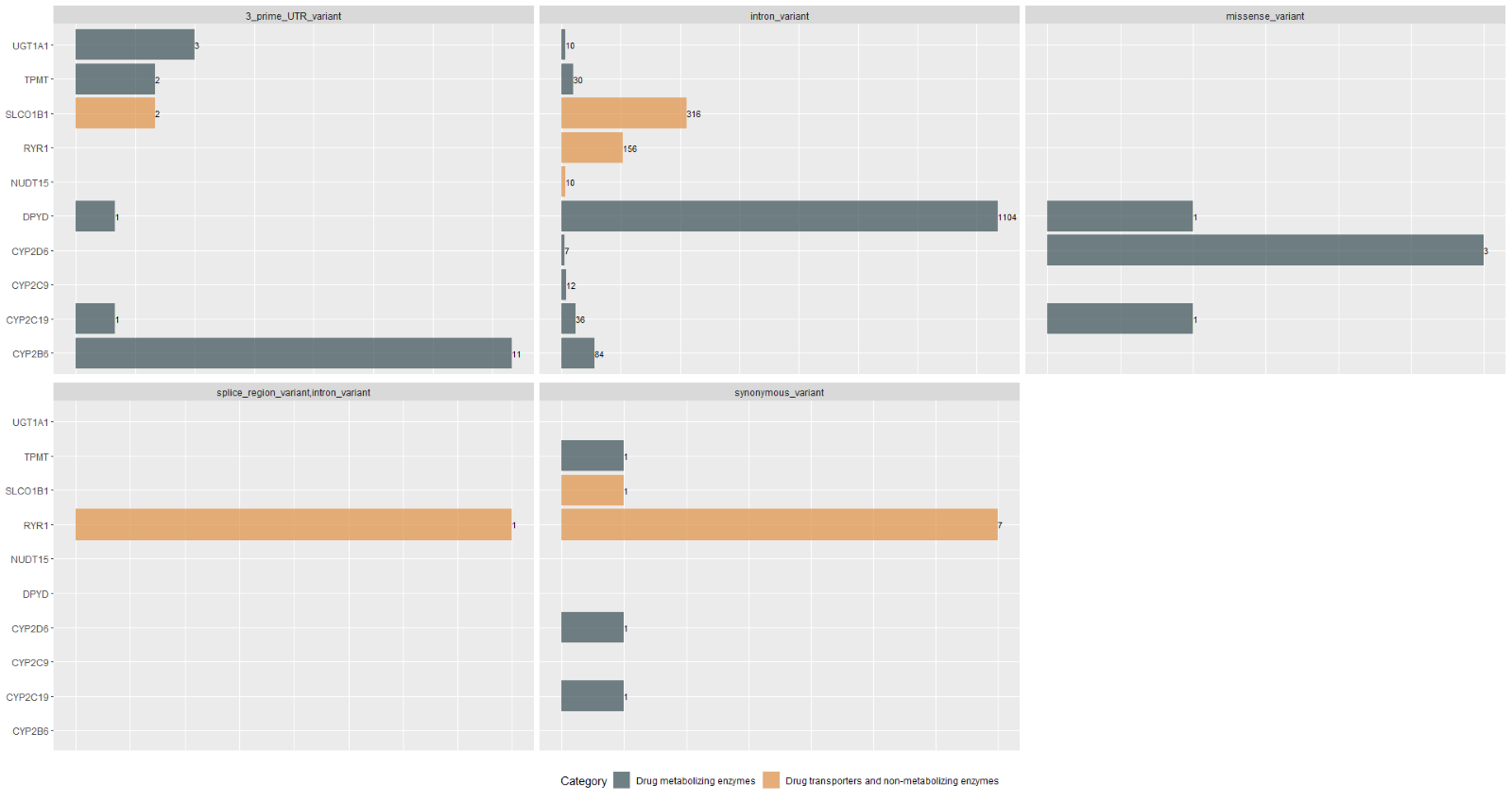


**Figure S2**. Distribution of PGx variants identified in the WGS data (first case study) that were not processed owing to many missing values. The graph presents the number of PGx variants, by gene, that were not processed any further by the machine learning model, according to the VEP consequence (i.e., 3’ UTR variant, intronic variant, missense variant, splice region variant and synonymous variant). The pharmacogenes are color-coded according to the corresponding PGx group: genes encoding drug metabolizing enzymes or genes encoding drug transporters or other non-metabolizing enzymes.

 
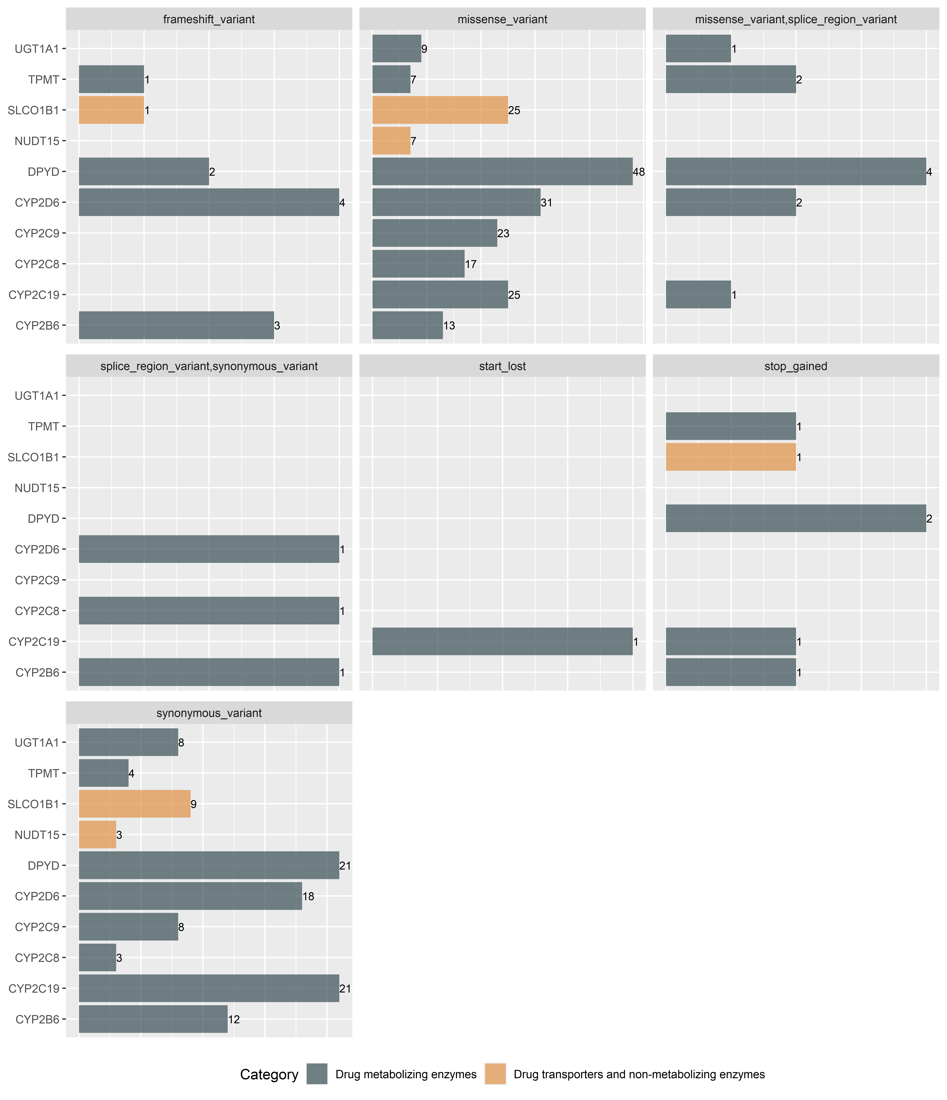


**Figure S3.** Sequence ontology consequences for the identified PGx variants, as derived from a Greek cohort of 304 individuals with psychiatric disorders (second case study). 343 PGx variants within the pharmacogenes of interest were identified in this cohort. Amongst the consequences are ‘frameshift’, ‘missense’, ‘missense or splice region’, ‘splice region’, ‘start lost’, ‘stop gained’ and ‘synonymous’ variants.

**Supplementary Table S1.** List of the represented pharmacogenes, which were included in the training dataset of the assessed machine learning models (AdaBoost, Multinomial logistic regression, Random Forest, XGBoost).

| **Pharmacogene** | **Description (HGNC)** | **Category** |
| --- | --- | --- |
| ***CYP2B6*** | Cytochrome P450 family 2 subfamily B member 6 | drug-metabolizing enzymes |
| ***CYP2C9*** | Cytochrome P450 family 2 subfamily C member 9 | drug-metabolizing enzymes |
| ***CYP2C19*** | Cytochrome P450 family 2 subfamily C member 19 | drug-metabolizing enzymes |
| ***CYP2D6*** | Cytochrome P450 family 2 subfamily D member 6 | drug-metabolizing enzymes |
| ***CYP2C8*** | Cytochrome P450 family 2 subfamily F member 8 | drug-metabolizing enzymes |
| ***DPYD*** | Dihydropyrimidine dehydrogenase | drug-metabolizing enzymes |
| ***UGT1A1*** | UDP glucuronosyltransferase family 1 member A1 | drug-metabolizing enzymes |
| ***NUDT15*** | Nudix hydrolase 15 | drug transporters and  non–drug metabolizing enzymes |
| ***RYR1*** | Ryanodine receptor 1 | drug transporters and  non–drug metabolizing enzymes |
| ***SLCO1B1*** | Solute carrier organic anion transporter  Family member 1B1 | drug transporters and  non–drug metabolizing enzymes |
| ***TPMT*** | Thiopurine S-methyltransferase | drug-metabolizing enzymes |

**Table S2.** Summary of the parameters and metric values for the tree-based models (AdaBoost, Random Forest, XGBoost), as tested in the present study. Parameters denoted with an asterisk (*) were tuned according to the achieved accuracy.

| **Approach** | **Parameters** | **Selected values** |
| --- | --- | --- |
| **Random Forest** | Number of trees (ntree)  *Number of variables randomly sampled as candidates at each split (mtry) | 1,000  2 |
| **AdaBoost** | Maximum tree depth (maxdepth)  *Number of trees (mfinal)  *Coefficient type (coeflearn) | 5  1,000  Zhu |
| **XGBoost** | *Boosting Iterations (nrounds)  Maximum Tree Depth (max_depth)  *Shrinkage (eta)  Minimum Loss Reduction (gamma)  Subsample Ratio of Columns (colsample_bytree)  Minimum sum of Instance Weight (min_child_weight)  Subsample percentage (subsample) | 150  5  0.1  0  0.8  1  1 |
